# Expression of Adipose Tissue Extracellular Matrix-Related Genes Predicts Weight Loss after Bariatric Surgery

**DOI:** 10.3390/cells12091262

**Published:** 2023-04-26

**Authors:** Óscar Osorio-Conles, Romina Olbeyra, Josep Vidal, Ainitze Ibarzabal, José María Balibrea, Ana de Hollanda

**Affiliations:** 1 Centro de Investigación Biomédica en Red de Diabetes y Enfermedades Metabólicas Asociadas (CIBERDEM), Instituto de Salud Carlos III (ISCIII), Monforte de Lemos Ave. 3-5, 28029 Madrid, Spain; 2 Institut d’Investigacions Biomèdiques August Pi i Sunyer (IDIBAPS), Rosselló Street 149, 08036 Barcelona, Spain; 3 Obesity Unit, Endocrinology and Nutrition Department, Hospital Clínic de Barcelona, Villarroel Street 170, 08036 Barcelona, Spain; 4 Gastrointestinal Surgery Department, Hospital Clínic de Barcelona, Villarroel Street 170, 08036 Barcelona, Spain; 5 Centro de Investigación Biomédica en Red Fisiopatologia de la Obesidad y Nutrición (CIBEROBN), Instituto de Salud Carlos III (ISCIII), Monforte de Lemos Ave. 3-5, 28029 Madrid, Spain

**Keywords:** extracellular matrix, weight loss, bariatric surgery, collagen 5, collagen 6, white adipose tissue

## Abstract

Background: We evaluated the association between white adipose tissue parameters before bariatric surgery (BS) and post-surgical weight loss, with an especial focus on extracellular matrix (ECM) gene expression. Methods: Paired samples from subcutaneous (SAT) and visceral adipose tissue (VAT) were obtained from 144 subjects undergoing BS. The association between total body weight loss (%TBWL) at 12 months after BS and the histological characteristics and gene expression of selected genes in SAT and VAT was analyzed. Results: Fat cell area, size-frequency distribution, and fibrosis in SAT or VAT prior to surgery were not associated with %TBWL. On the contrary, the SAT expression of *COL5A1* and *COL6A3* was associated with %TBWL after BS (both *p* < 0.001), even after adjusting for age, gender, baseline BMI, and type 2 diabetes status (T2D). Furthermore, in logistic regression analyses, the expression of these genes was significantly associated with insufficient WL (IWL = TBWL < 20%) after BS (respectively, *p* = 0.030 and *p* = 0.031). Indeed, in ROC analysis, the prediction of IWL based on sex, age, BMI, T2D, and the type of surgery (AUC = 0.71) was significantly improved with the addition of SAT-*COL5A1* gene expression (AUC = 0.88, Z = 2.13, *p* = 0.032). Conclusions: Our data suggest that the expression of SAT ECM-related genes may help explain the variability in TBWL following BS.

## 1. Introduction

Bariatric surgery (BS) is currently the most effective intervention to help subjects with morbid obesity achieve and sustain significant weight loss (WL) [1,2]. However, heterogeneity exists in WL trajectories among patients following BS [3], with as many as 25% of bariatric patients presenting with poor WL after BS [4]. Surgical, psychological, and biological mechanisms have been suggested as predictors of the variable WL response after BS [5]. Nevertheless, the biological underlying mechanisms have not been fully elucidated.

White adipose tissue (WAT) is a hallmark of obesity and a highly dynamic organ. Thus, it has been proposed that certain features and the remodeling of WAT could be associated with variability in the health outcomes that ensue following BS [6,7,8,9]. It has been proposed that “healthy” WAT expansion upon positive energy balance is the result of the orchestrated modulation of inflammatory phenomena, extracellular matrix (ECM) remodeling, and angiogenesis [10]. Notably, it has been shown that the WAT accumulation of ECM proteins, resulting in fibrosis prior to surgery, is associated with lesser WL at 12 months after surgery [7]. Furthermore, it has been suggested that the inappropriate remodeling of the ECM may result in adipocyte mechanical stress leading to limited adipocyte shrinkage upon negative balance [9].

Against this background, in the present study, we aimed at further evaluating the association between WAT parameters at baseline and WL following BS, with an especial focus on not only fibrosis but also the gene expression of the WAT ECM matrix.

## 2. Materials and Methods

### 2.1. Study Participants

All study participants (*n* = 144) were selected among those who underwent Roux-en-Y gastric bypass (RYGB) or sleeve gastrectomy (SG) as their primary BS at our institution between April 2018 and July 2021. The patients ensured a minimum follow-up period of 1 year after surgery and gave signed informed consent to participate in the SAT and VAT sample collection. Eligibility criteria for BS were age between 18 and 70 years and BMI above 40 Kg/m^2^ or above 35 Kg/m^2^ if obesity-related comorbidities were present [11]. The technical aspects and selection criteria for RYGB or SG at our institution have previously been reported [12,13]. Patients with history of malignancy, a diagnosis of chronic inflammatory disease, active infectious disease, or drug abuse or daily alcohol consumption > 20 g were excluded. To test the association between gene expression and weight loss, BS patients were divided into an exploratory (*n* = 75) and a validation cohort (*n* = 39). Baseline anthropometric and clinical data of the exploratory and validation cohorts are summarized in Supplementary Table S1.

Additionally, SAT mRNA levels of specific ECM components were determined in 17 control subjects with no history of obesity and in 18 ex-obese patients (all BMI < 30 kg/m^2^). Control patients were selected among those undergoing abdominal surgery for benign processes (cholecystectomy (*n* = 9), eventroplasty (*n* = 6), umbilical hernia repair (*n* = 1), or appendectomy (*n* = 1)) at our institution. Ex-obese patients were selected among subjects with prior BS that underwent exploratory laparoscopy (*n* = 17) or cholecystectomy (*n* = 1). Of them, 17 had received RYGB and 1 had received SG at 1 (*n* = 13), 2 (*n* = 1), 3 (*n* = 1), 4 (*n* = 1) and 10 years (*n* = 2) prior to AT sample collection. Anthropometric and clinical data of these groups are summarized in Supplementary Table S2.

The Research Ethics Committee approval conforming to the Declaration of Helsinki for sample collection was obtained from the Clinical Research Ethics Committee (CEIC) of Hospital Clinic de Barcelona (R120615-084, 13 October 2016).

### 2.2. Anthropometric and Clinical Assessments

Anthropometric measurements were collected in the same consultations before surgery and throughout the follow-up period, following standardized procedures. Body weight (BW) loss was expressed as a percentage of total body weight loss relative to baseline body weight (%TBWL = 100 * (baseline BW−current BW)/baseline BW). Insufficient weight loss (IWL) was defined as TBWL < 20% [14]. Hematological and biochemical parameters were determined at the Core Laboratory of the Biomedical Diagnostic Center using an Advia 2400 analyzer (Siemens Healthcare S.L.U., Getafe, Spain). The presence of T2D was considered in subjects who had either fasting plasma glucose (FPG) ≥ 126 mg/dL or glycosylated haemoglobin (HbA1c) ≥ 6.5% or were on antidiabetic treatment.

### 2.3. Tissue Processing and Histology

At the time of BS, paired VAT and SAT samples were collected in DMEM and rinsed in PBS. A portion was immediately frozen before RNA analysis. Another portion was fixed overnight at 4 °C in 4% paraformaldehyde and processed for standard paraffin embedding. Starting at the tissue apex, 3 × 3 μm sections were made at a minimum of 100 μm intervals across the sample tissue. Hematoxylin and eosin staining was used to measure adipocyte areas of at least 3000 cells per sample from randomly selected fields at 4× magnification using Adipocytes Tools, an ImageJ macro-based algorithm for ImageJ software (National Institutes of Health, Bethesda, MD, USA; http://imagej.nih.gov/ij/ [accessed on 24 April 2023]). Adipocyte average area was calculated, and the frequency distribution analysis of fat cells separated into bin intervals of 200 µm^2^ and their representative sizes was performed. Sirius red staining was used for quantification of fibrosis [15]. Automated analysis has been carried out using MRI Fibrosis Tool, an ImageJ macro-based algorithm. Total fibrosis was quantified at 4× magnification in six random fields on the edge of the fat lobule for each biopsy and expressed as a percentage of fibrous tissue area stained with Sirius red/total tissue surface within the image. Pericellular fibrosis—around the cells—was quantified at 10× magnification in six random fields inside the fat lobule for each biopsy and expressed as a percentage of Sirius red staining/total tissue surface. Digital images were captured under an Olympus × 600 microscope (Olympus Corporation, Tokyo, Japan). Additionally, determinations of hydroxyproline residues after acid hydrolysis of collagen, as a measure of total collagen, were performed in SAT lysates using a colorimetric hydroxyproline assay kit (Ab222941, Abcam, Cambridge, UK). Absorbance was measured at 560 nm in an Infinite M Plex plate reader (Tecan, Männedorf, Switzerland).

### 2.4. RNA Extraction and Quantitative Real-Time PCR

Tissue samples were immediately frozen before RNA analysis. Total RNA was isolated using RNeasy Lipid Tissue Mini Kit (Qiagen, Hilden, Germany). Concentration and purity were measured using a NanoDrop 1000 spectrophotometer (Thermo Scientific, Waltham, MA, USA). Equal amounts of RNA from SAT and VAT were reverse transcribed using the Superscript III RT kit and random hexamer primers (Invitrogen, Carlsbad, CA, USA). Reverse transcription reaction was carried out for 90 min at 50 °C and for an additional 10 min at 55 °C. An expression analysis of 80 genes in WAT inflammation, adipogenesis, autophagy, fatty acid metabolism and oxidation, thermogenesis and glucose metabolism, cytokines, adipokines, and ECM was performed in both fat depots. Real-time quantitative PCR (qPCR) was performed with a 7900HT Fast Real-Time PCR System (Applied Biosystems, Foster City, CA, USA) using GoTaq^®^ qPCR Master Mix (Promega Biotech Ibérica, Madrid, Spain). Expression relative to the house-keeping gene *RPL6* was calculated using the delta Ct (DCt) method. Gene expression is presented as the 2^(−DCt)^ values. The list of primers used in this study is provided in Supplementary Table S3.

### 2.5. Statistics

Continuous data with normal and non-normal distribution is expressed with arithmetic means and standard deviations (SD) or with medians and 95% confidence interval (95% CI), respectively. Categorical variables are expressed with frequencies and proportions. Normality assumption was tested with D’Agostino–Pearson omnibus normality test. Mann–Whitney U test, Student’s t-test, or Fisher’s exact test was used when adequate to assess the magnitude of the difference between groups. One-way ANOVA or Kruskal–Wallis tests, followed by Tukey’s or Dunn’s multiple comparisons tests, respectively, were used to compare the magnitude of the difference among the three study groups when adequate. Pearson’s r or Spearman’s rho were used to test for correlations. Univariate and multivariable linear general models were used to evaluate the independent effect of variables of interest on TBWL. Logistic regression models were used to test the effect on IWL. Outliers were identified using the ROUT (robust regression and outlier removal) method with a coefficient Q = 1% and removed, with the exception of non-obese and ex-obese groups. Receiver operating curve (ROC) analyses were developed by using the probability function determined by the logistic regression models, and the area under the curve (AUC) was calculated for each ROC curve. AUCs (95% CI) from different ROC curves were compared using a Z test [16].

In all cases, a two-tailed *p*-value < 0.05 was considered statistically significant. GraphPad PRISM (GraphPad Software, version 6.0, San Diego, CA, USA) and Statistical Package for Social Sciences software (SPSS, version 17.0, Chicago, IL, USA) were used to perform the analyses.

## 3. Results

### 3.1. White Adipose Tissue Parameters and Weight Loss after BS

The baseline anthropometric and clinical data of subjects who underwent BS are summarized in Table 1. The data on BW is available for the 4-, 8-, and 12-month follow-ups of 144 (100%) subjects, for the 24-month follow-ups of 117 (81.2%) subjects, and for the 36-month follow-ups of 34 (23.6%) subjects. The mean %TBWL at the 4-, 8-, 12-, 24-, and 36-month time points was 19.8 ± 6.1%, 28.5 ± 6.9%, 31.2 ± 7.6%, 30.3 ± 9.4%, and 28.2 ± 12%, respectively. The percentage of participants with IWL during the follow-up period was 10.4% (*n* = 15) at 12 months, 11.1% (*n* = 13) at 24 months, and 23.5% (*n* = 8) at 36 months. As the occurrence of follow-up at 12 months after surgery was 100%, for the analysis on the association between AT features and %TBWL, we considered the data from this time point.

**Figure 1:**
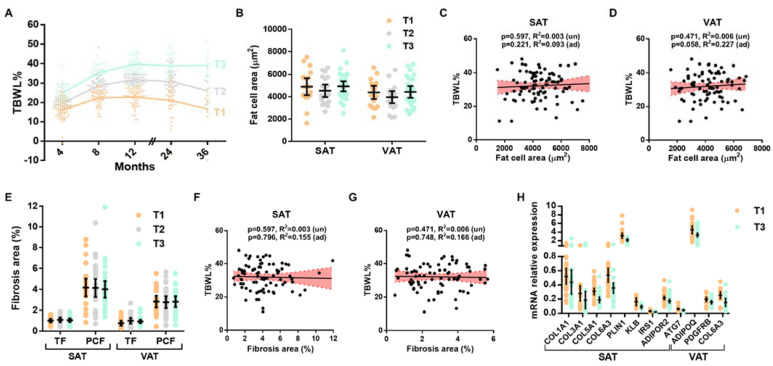
Adipose tissue parameters and their association with weight loss. (**A**) Weight loss trajectories of study patients divided into tertiles of TBWL at 12 months. (**B**) Comparison of mean fat cell areas ± SD between tertiles of TBWL (*n* = 87). (**C**,**D**) Regression analysis between TBWL and SAT (**C**) and VAT (**D**) fat cell areas. (**E**) Comparison of mean total (TF) and pericellular fibrosis (PCF) areas ± SD between tertiles of TBWL (n=87). (**F**,**G**) Regression analysis between TBWL and pericellular fibrosis area in SAT (**F**) and VAT (**G**). (**H**) Differently expressed genes in adipose tissue from tertiles 1 and 3 of TBWL (*n* = 50). Scatterplots present crude, non-adjusted relationships. Simple linear regression (solid lines) and 95% confidence interval (dashed lines) are shown and *p*-values are unadjusted (un) or adjusted for sex, age, baseline BMI, T2D, and type of surgery (ad).

The tertiles of TBWL% at 12 months (T1–3) are shown in Figure 1A. The median (95% CI) values of %TBWL at this time point were 23.39 (22.93–24.73), 31.35 (30.73–32.78), and 39.43 (37.84–40.28) for the T1, T2, and T3 tertiles, respectively (Dunn’s test *p* < 0.0001 for all comparisons). The baseline average fat cell areas of SAT and VAT were comparable among TBWL tertile groups (Figure 1B). Similarly, frequency distribution analyses showed no significant differences in either depot among TBWL tertile groups when fat cell areas were divided by size into the bin intervals of 200 µm^2^ or into three representative sizes (Supplementary Figure S1). In a regression analysis, the mean fat cell sizes of SAT or VAT were not associated with TBWL even after adjusting for sex, age, baseline BMI, T2D, and the type of surgery (Figure 1C,D).

The degree of histological fibrosis measured by picrosirius red staining is shown in Figure 1E and Supplementary Figure S2. Total and pericellular fibrosis were comparable among groups in both fat depots (all *p* > 0.05). No association between TBWL% and pericellular fibrosis was found even after adjusting for covariates (all *p* > 0.05, Figure 1F,G). This was also the case for total fibrosis. In a subset of patients, determinations of hydroxyproline residues were performed in samples from SAT. Again, no differences among WL tertiles or an association with %TBWL were found (Supplementary Figure S2C,D).

Finally, the expression analysis of 80 genes in WAT samples from the T1 and T3 tertiles of TBWL in the exploratory cohort showed that the expression of 12 genes, 9 in SAT and 3 in VAT, was significantly different among these tertiles (Figure 1H). SAT expressions of alpha 1 chains of collagens I (*COL1A1*), III (*COL3A1*), and V (*COL5A1*); perilipin 1 (*PLIN1*); klotho beta (*KLB*); insulin receptor substrate (*IRS1*); adiponectin receptor 2 (*ADIPOR2*; and autophagy-related gene 7 (*ATG7*) were upregulated in subjects from T1. This group also showed higher VAT mRNA levels of adiponectin (*ADIPOQ*) and platelet-derived growth factor receptor beta (*PDGFRB*). The expression of the alpha 3 chain of collagen VI (*COL6A3*) was upregulated in both fat depots from subjects in T1.

### 3.2. Expression of ECM Components and Associations with Weight Loss

We then tested the linear association between these differently expressed genes and TBWL in the exploratory cohort. Five out of the twelve differently expressed genes were related to TBWL within the exploratory cohort, and all of them were differently expressed in SAT (Table 2). The association of *ADIPOR2* was lost after adjusting for covariates, while associations of *PLIN1* and *COL1A1* were only significant after adjusting for age, sex, BMI, the type of surgery, and T2D. The SAT expression of *COL5A1* and *COL6A3* remained significantly associated with TBWL both before and after adjustment. Although weight loss was lesser in the validation cohort than in the exploratory cohort (Table S1), the associations between the alpha 1 chains of the three collagens remained significant in the validation cohort and were, thus, included in the subsequent analysis.

**Figure 2:**
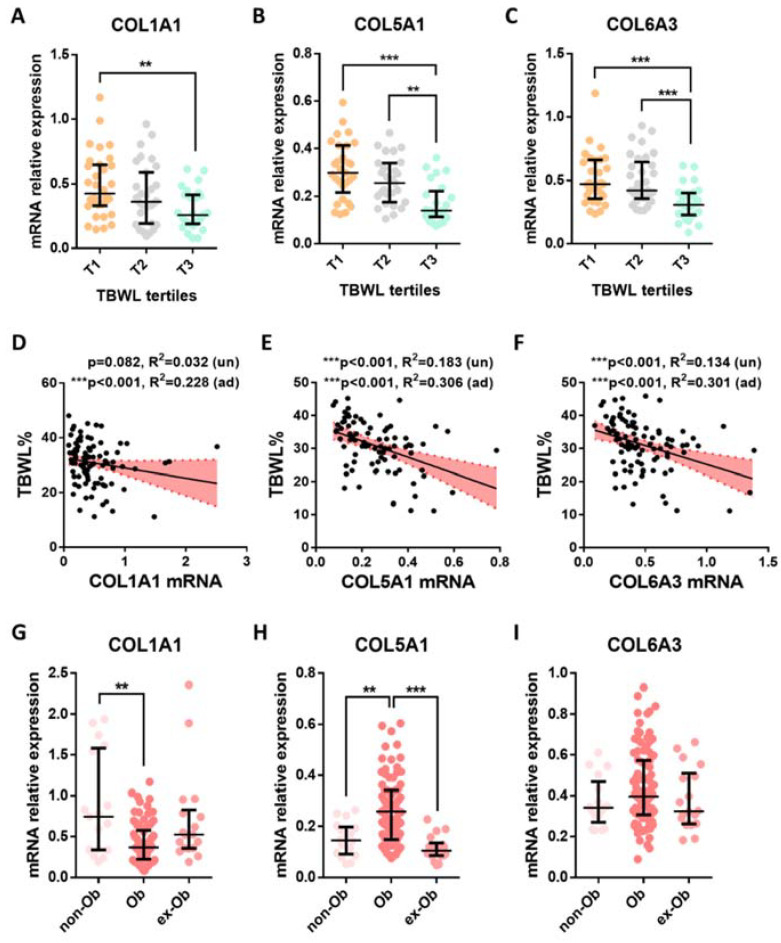
Association of collagen mRNA levels with TBWL and BMI. (**A**–**C**) Comparison of gene expression levels in SAT between TBWL tertiles. (**D**–**F**) Simple linear regression (solid lines) and 95% confidence interval (dashed lines). *P*-values are unadjusted (un) or adjusted for sex, age, baseline BMI, T2D, and type of surgery (ad). (**G**–**I**) mRNA levels in subjects with obesity (Ob) and without obesity (non-Ob) and in ex-obese individuals (ex-Ob). Data are presented as scatterplots and mean ± SD. ** = *p*< 0.01, *** = *p* < 0.001.

The gene expression levels of *COL1A1*, *COL5A1*, and *COL6A3* were significantly modified across the TBWL tertiles in the pooled cohort (ANOVA *p* < 0.005 for each, Figure 2A–C). In addition, *COL5A1* and *COL6A3* were downregulated in the T3 tertile compared to the T2 and T1 tertiles (*p* < 0.005). Next, we evaluated the addition of the mRNA levels of these genes in the performance of a clinical model predicting TBWL. A model composed by main clinical variables (sex, age, baseline BMI, T2D, and the type of surgery) was able to explain a 13.4% of TBWL in our cohort. Although, as stated above, *COL1A1* mRNA levels were only associated with TBWL after adjusting for covariates (Figure 2D); its addition significantly increased the performance of the model (adjusted R^2^ = 0.228, F change *p* = 0.005). The *COL5A1* and *COL6A3* mRNA levels independently explained a 18.3% and 13.4% reduction (Figure 2D,F), and their addition to the clinical model significantly improved the amount of TBWL explained (R^2^ = 0.306 and R^2^ = 0.301, respectively, F change *p* < 0.001 for both). A summary of these regression models is shown in Supplementary Table S4.

Gene expression levels of these ECM components were also measured in groups of ex-obese subjects and never-obese subjects (Figure 2G–I). Interestingly, although the SAT expression of *COL1A1* and *COL5A1* differed across study groups (ANOVA *p* < 0.005 and *p* < 0.0001, respectively), they showed different patterns. While *COL1A1* was downregulated in Ob versus never-Ob subjects, *COL5A1* was significantly upregulated in Ob versus never- and ex-Ob patients. On the other hand, *COL6A3* mRNA levels were comparable between study groups (ANOVA *p* = 0.151), even if non- and ex-Ob were taken together.

The expression of these three genes was highly correlated with the non-Ob and Ob groups with a Spearman’s rho between 0.47 and 0.83 (*p* < 0.001 for all), while only the association between *COL5A1* and *COL6A3* was significant within the ex-Ob group (r = 0.58, *p* = 0.01). Taken together and irrespective of study group, all gene expressions were highly correlated with each other. In addition, relevant correlations with clinical variables were found. *COL1A1* was negatively associated with fasting plasma glucose (r = −0.21, *p* = 0.014); *COL5A1* was related to BMI (r = 0.478, *p* < 0.0001), fasting plasma glucose (r = 0.194, *p* = 0.035), AST (r = −0.189, *p* = 0.048) and TG levels (r = 0.196, *p* = 0.038); and *COL6A3* was associated with BMI (r = 0.275, *p* = 0.001). Interestingly, *COL6A3* showed an opposite association with HbA1c in Ob (r = −0.215, *p* = 0.033) and ex-Ob groups (r = 0.545, *p* = 0.031). Notably, *COL6A3* was correlated with pericellular fibrosis in SAT from Ob subjects in an inverse manner (r = −0.37, *p* = 0.009).

### 3.3. Predictive Value of ECM Components on Insufficient Weight Loss

**Figure 3:**
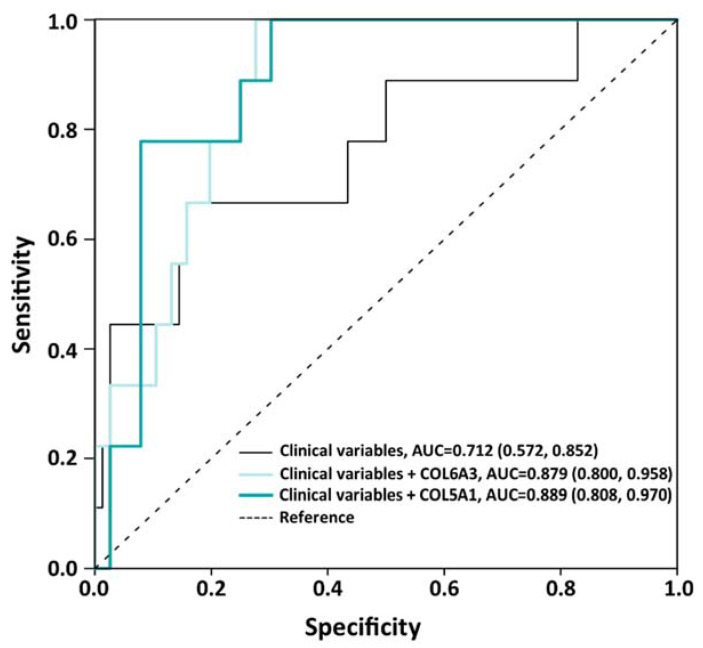
ROC curves of the clinical and combined models for the prediction of IWL.

In the logistic regression analyses, the expressions of *COL5A1* and *COL6A3* were significantly associated with IWL at 12 months after BS with β: 112.75, 95% CI: 1.58–804.82 (*p* = 0.030) and β: 11.34, 95% CI: 1.24–103.58 (*p* = 0.031), respectively, while *COL1A1* mRNA levels were not (*p* = 0.745). Finally, ROC analyses were performed to assess the addition of *COL5A1* or *COL6A3* mRNA levels on the accuracy of a clinical model predicting IWL after BS (Figure 3). The AUC for a clinical model composed of sex, age, BMI, baseline T2D, and the type of surgery was 0.71. The addition of *COL6A3* gene expression to the model increased the AUC up to 0.87, although the change was statistically non-significant (Z = 1.91, *p* = 0.055). On the contrary, the addition of *COL5A1* gene expression significantly increased the performance of the model (AUC = 0.88, Z = 2.13, *p* = 0.032).

## 4. Discussion

In this study, we evaluated potential associations between WAT parameters and TBWL at 1 year in a population of subjects with severe obesity that had undergone BS. While no differences in WAT histology were found, we identified a set of genes that were differently expressed in subjects in the upper- or lower-tertile of TBWL. We tested the linear association of these candidate genes with TBWL after adjusting for covariates. A negative association for three ECM components in SAT was established across an exploratory and a validation cohort. Among these genes, *COL5A1* mRNA levels was identified as an independent predictor of TBWL and increased the performance of a clinical model predicting IWL at 1 year after BS.

In a previous work, Muir et al. found a negative association between VAT adipocyte size and TBWL 1 year after BS [17]. Nevertheless, this association was lost after adjusting for age, HbA1c, and the type of surgery. Our results did not support a relation of fat cell size in either depot or TBWL after BS. In addition, data from three independent studies carried out by Karine Clément’s group identified the degree of fibrosis in SAT as an independent predictor of limited weight or fat mass loss after BS [15,18,19]. Again, our data failed to show a relationship between TBWL and the degree of fibrosis in SAT evaluated either by histological picrosirius red staining or by the biochemical determination of hydroxyproline residues. It could be argued that differences in sample size and patient characteristics (higher age and a lower baseline BMI in our population) might contribute to these disagreements.

A number of associations between changes in SAT expression of genes and BW evolution after diet-induced WL have been previously reported [20,21,22,23]. Thus, changes in the expression of genes implicated in cell stress [20,21,24], cellular growth and proliferation [22], cell death, mitochondrial oxidative phosphorylation [22,23], citric acid cycle [23], or lipid transport and metabolism [23,24] have been associated with the amount of WL. Positive correlations between expression and DNA methylation have also been reported for a number of genes [24]. Nevertheless, reports relating changes in ECM-related genes and WL are scarce [24] and few relationships have been established between baseline WAT transcriptome and WL after BS.

In an exhaustive microarray study, Kim et al. correlated perioperative VAT expression of genes implicated in lipid and glycerolipid metabolism, neuronal function, and apoptosis with WL after RYGB, although the findings were drawn from a small sample size [25]. In addition, Lasselin et al. found that the higher VAT gene expression of IL-10 and M1-macrophage markers before BS predicts poorer BMI reduction after surgery [26]. Both VAT and, in particular, SAT levels of protein glycoxidation have also been directly correlated with WL after BS [27]. Notably, in another study performed only on females, baseline *UCP2* and *PLIN1* expression in SAT were negatively associated with TBWL at 6 months after surgery [28]. In our study, *PLIN1* was found inversely related to TBWL in the exploratory cohort but then lost in the validation cohort and, thus, was not selected for further analysis. The inclusion of males in our study may partly explain such differences. Finally, a circulating miRNAs signature has been identified to predict WL effectiveness following BS [29]. These three miRNAs were implicated in adipogenesis, oxidative stress, and energy expenditure.

Starting from 12 differently expressed genes between the T1 and T3 tertiles of WL, we validated 3 genes in SAT coding for ECM components that were associated with TBWL. Notably, only three out of the six tested genes coding for collagen chains were identified as significant predictors of TBWL in our study. In addition, the baseline expression of any other gene coding for ECM components—hyaluronan synthases, elastin, fibronectin, and biglycan—or ECM modifiers—hyaluronidases (HYALs), lysyl oxidases (LOXs), and matrix metalloproteinases (MMPs) and their inhibitors (TIMPs)— showed a relation to the amount of WL. Thus, only 3 out of 24 genes related to ECM were associated with TBWL in our population. Furthermore, these genes showed a divergent modulation in obesity, with only *COL6A3* being correlated to the degree of fibrosis, and, notably, in an inverse manner.

Adipose tissue ECM dynamics during WL are incompletely understood. Previous studies have proposed that increased fibrosis in SAT prior to surgery negatively affects the ability to lose weight after BS [15,18]. While some authors found that fibrosis levels remained unchanged after BS [30], increased collagen deposition has been reported by others [31]. Similarly, specific sets of ECM components were found up- or downregulated after surgery [31,32]. Interestingly, despite increased Sirius red staining, decreased ECM cross-linking and increased collagen degradation were found [31]. A hypothesis of how ECM might modulate fat mass loss has been proposed by Mariman et al. [33]. During a weight loss phase, adipocytes shrink in volume [34,35]. If this process is not accompanied by an adaptation of the surrounding ECM, traction forces can occur, leading to cellular stress that might promote the restoration of its original fat content [36]. Taken together, these results suggest that ECM composition and dynamics, more than its abundance, merits further attention when evaluating the relationship between adipose ECM and WL.

Collagens, in addition to the most abundant structural components of the ECM, are also implicated in cell adhesion, migration, differentiation, and morphogenesis [37]. Collagen I, the most abundant ECM molecule [38], is present together with types IV, V, and VI in WAT-ECM, with collagen VI being highly enriched [39]. In our study, *COL5A1* and *COL6A3* were associated with BMI, while a decreased expression of *COL1A1* was found in Ob versus non-Ob patients, in accordance with previous works from others [40,41,42,43]. These three genes were found to be increased in SAT together with a strong reduction in inflammation 1 year after biliopancreatic diversion with duodenal switch [32], but the authors did not evaluate the predictive value of baseline expression on the WL response, possibly because of the small sample size.

Notably, only *COL5A1* and *COL6A3* were associated with TBWL, before and after adjusting for covariates, and predicted IWL. The knock-out of collagen VI in a mouse model resulted in the uninhibited expansion of individual adipocytes yet the absence of metabolic derangements associated with the obese phenotype [44]. Among these chains, alpha 3 chain has been the most extensively studied in WAT and associations with human obesity and insulin resistance have been reported [40,45], yet not all in the same direction [46]. The signaling role of endotrophin, a cleavage product of the type VI alpha 3 chain that can be released into the circulation, has received great attention in recent years [47,48,49,50,51].

Here, we report an inverse correlation between *COL6A3* and HbA1c among Ob and ex-Ob groups. While a negative association has been previously found in obesity [46], this alternate relation in lean versus obese subjects has never been reported to our knowledge. While a deleterious role of endotrophin on insulin sensitivity was found in mouse models [51], how *COL6A3* is processed to produce endotrophin in the ECM, or whether the cleavage process is regulated, remains poorly understood. *COL6A3* was found upregulated after a 1-year WL intervention consisting of a very-low-energy diet followed by a normal WL diet in conjunction with exercise promotion, and this correlated with changes in gene DNA methylation [24]. Notably, McCulloch et al. showed that diet and surgery-induced weight loss increase *COL6A3* expression in SAT [46]. Nevertheless, the association between *COL6A3* and WL after surgery is novel. Potential functional relationships between COL6a3/endotrophin and BS outcomes merit further evaluation.

Interestingly, only the addition of *COL5A1* significantly increased the AUC of a clinical model predicting IWL at 12 months after BS in the ROC analysis. Haploinsufficiency of the *COL5A1* gene, which encodes the proα1(V) chain of type V collagen, is the classical form of the Ehlers–Danlos syndrome, a connective tissue disorder characterized by skin hyperextensibility, atrophic scarring, and generalized joint hypermobility [52,53]. Consequently, polymorphisms that confer an increased range of motion [54] and decreased tendon-ligament injuries have been found for this gene [55]. To date, *COL5A1* has also been implicated as an oncogenic protein in the central nervous system [56,57] and in gastric [57,58], ovarian [57,59], breast [57,60], and many other cancers, being negatively related to the prognosis of 11 cancers [56,57,60].

Type V collagen has been previously found to be upregulated in SAT from lean versus obese patients and colocalized with fibrotic areas and large blood vessels, while its addition in angiogenesis assays was shown to inhibit endothelial budding, suggesting an an inhibitory role in angiogenesis [61]. In addition, these authors found an inverse association with insulin sensitivity; however, in this sense, divergences by ethnicity have been also identified in women [62]. Despite the fact that insulin levels were not measured in our study, we found a positive correlation between *COL5A1* and fasting plasma glucose. Collagen V is produced by preadipocytes [63] and promotes WAT hyperplasia [64], which, together with type VI collagen, shows specific dynamics during adipogenesis and may play a role in fat lobule organization [65]. Deciphering whether its implication in WAT structure or angiogenesis is responsible for its predictive value on WL outcomes after BS warrants further investigation.

We acknowledge our study is not without limitations. First, the WAT samples were collected only prior to surgery, and the potential changes in ECM composition after BS were not evaluated. Thus, BS-induced changes in WAT ECM composition or the possible effects of ECM dynamics on WL response could not be ascertained. Second, gene expression at protein level was not determined, preventing associations between the content of these proteins and the WL response. Finally, this is an observational study and mechanistic insights are not provided.

## 5. Conclusions

Taken together, our data suggest the expression of SAT extracellular matrix-related genes may help explain the variability in TBWL following commonly performed BS techniques such as RYGB and SG. Furthermore, among those genes, the expression of *COL5A1* may help predict TBW < 20% after BS. We deem our data suggest additional studies are needed to further demonstrate the role of ECM composition and dynamics as determinants of the clinical outcomes of weight loss interventions.

## Figures and Tables

**Table 1:** Baseline characteristics of the study patients undergoing bariatric surgery.

	Baseline (*n* = 144)
Type of Surgery (RYGB)	88 (61.1%)
Sex (Female)	124 (86.1%)
Age (years)	51.8 ± 10.9
Weight (kg)	113.8 ± 25.7
BMI (kg/m^2^)	43.1 (42.1, 44.5)
T2D (yes/no)	49 (34.0%)
T2D treatment (yes/no)	48 (33.3%)
T2D medications (number)	0 (0–0)
Insulin treatment (yes/no)	19 (13.2%)
Hypertension (yes/no)	47.9 (32%)
Statins (yes/no)	49 (34.0%)
FPG (mg/dL)	99 (96, 102)
HbA1c (%)	5.7 (5.6, 5.8)
Total cholesterol (mg/dL)	184.6 ± 36.18
HDL-cholesterol (mg/dL)	
Male	38.5 (37, 44)
Female	50.55 ± 10.65
LDL-cholesterol (mg/dL)	
Male	108.9 ± 33.8
Female	111.7 ± 27.08
Tryglycerides (mg/dL)	
Male	130.5 ± 55.9
Female	116 (106, 130)
hs-CRP (mg/L)	0.66 (0.58, 0.85)
AST (UI/L)	20 (19, 22)
ALT (UI/L)	23 (21, 25)
GGT (UI/L)	26 (24, 28)

Data are presented as mean  ±  SD, median (95% CI), or percentage (%). BMI, body mass index; T2D, type 2 diabetes; FPG, fasting plasma glucose; HbA1c, A1c glycosylated haemoglobin; HDL, serum high-density lipoprotein; LDL, serum low-density lipoprotein; hs-CRP, high-sensitivity C-reactive protein; AST, serum aspartate aminotransferase; ALT, serum alanine aminotransferase; GGT, gamma-glutamyl transferase; TBWL, total body weight loss.

**Table 2:** Regression analysis between gene expression levels in SAT and TBWL.

	Exploratory		Validation		Pooled	
	β (95% CI)	*p*	β (95% CI)	*p*	β (95% CI)	*p*
*PLIN1*						
Unadjusted	−3.44 (−7.12, 0.29)	0.069	−0.791 (−2.33, 0.75)	0.305	−1.37 (−2.8, 0.07)	0.061
Adjusted ^1^	−3.52 (−6.94, −0.11)	0.044	−0.72 (−2.14, 0.71)	0.312	−1.35 (−2.69, −0.01)	0.049
*ADIPOR2*						
Unadjusted	−21.71 (−42.91, −0.50)	0.045	−21.02 (−49.59, 7.55)	0.145	−22.34 (−39.01, −5.48)	0.010
Adjusted ^1^	−16.86 (−36.62, 2.89)	0.093	−25.01 (−50.05, 0.019)	0.050	−20.02 (−35.76, −4.27)	0.013
*COL1A1*						
Unadjusted	−4.31 (−9.29, 0.67)	0.089	−8.51 (−18.46, 1.25)	0.085	−3.65 (−7.77, 0.47)	0.082
Adjusted ^1^	−5.87 (−10.44, −1.30)	0.013	−8.93 (−17.91, 0.045)	0.049	−5.25 (−8.99, −1.51)	<0.001
*COL6A3*						
Unadjusted	−11.87 (−21.05, −2.70)	0.012	−8.75 (−16.48, −1.01)	0.028	−11.40 (−17.37, −5.43)	<0.001
Adjusted ^1^	−13.23 (−20.73, −3.73)	0.006	−8.06 (−15.81, −0.31)	0.042	−12.25 (−17.68, −6.82)	<0.001
*COL5A1*						
Unadjusted	−24.61 (−41.31, −7.92)	0.005	−21.01 (−35.44, −6.58)	0.005	−23.59 (−34.18, −13.01)	<0.001
Adjusted ^1^	−24.79 (−43, −6.59)	0.009	−16.74 (−30.94, −2.53)	0.022	−23.78 (−34.7, −12.86)	<0.001

^1^ Multiple linear regression adjusted for age, sex, BMI, type of surgery, and T2D. β, unstandardized beta; CI, confidence interval.

## Data Availability

All data presented in this study are reported in this manuscript or available in the Supplementary Materials.

## Supplementary Materials

The following supporting information can be downloaded at: https://www.mdpi.com/article/10.3390/cells12091262/s1. Figure S1: Adipocyte size distribution across weight loss tertiles. Figure S2: Adipose tissue fibrosis across weight loss tertiles. Table S1: Clinical characteristics of the exploratory and validation cohorts. Table S2: Clinical characteristics of non-obese and ex-obese subjects. Table S3: List of primers used in this study. Table S4: Summary of multivariate regression models predicting TBWL.
