# Supplementary material for: Expression of Adipose Tissue Extracellular Matrix-Related Genes Predicts Weight Loss after Bariatric Surgery
Source: Cells. 2023 Apr 26;12(9):1262. doi: 10.3390/cells12091262 (PMC10177079; doi:10.3390/cells12091262)
Supplement: Supplementary file 1 [file cells-12-01262-s001.zip › cells-2247387-supplementary.pdf]

Supplementary Table S1. Clinical characteristics of the exploratory and validation cohorts.

	Exploratory cohort	Validation cohort	p	Pooled cohort
N	75	39		114
Females (%)	58(77)	33(85)	0.127	91(80)
Age	50.42 ± 10.10	51.82 ± 11.39	0.509	50.53 ± 10.65
BMI	43.43 (41.02, 47.91)	45.97 (40.35, 48.41)	0.565	43.49 (41.01, 48.18)
T2D (%)	21(28)	17(44)	0.155	38(33)
Type of surgery				
SG (%)	27(36)	15(39)	>0.999	42(37)
RYGB (%)	45(60)	24(61)		69(60)
TBWL (%)	31.98 ± 7.97	29.15 ± 6.35	**0.043**	31.25 ± 7.72

Data are presented as the mean ± SD, median (95%CI) or number (%). BMI, body mass index; T2D, type 2 diabetes; SG, sleeve gastrectomy; RYGB, Roux-en-Y Gastric Bypass; TBWL, total body weight loss.

Supplementary Table S2. Clinical characteristics of non-obese and ex-obese subjects.

	non-Ob (n=17)	ex-Ob (n=18)
**Sex (Female)**	13 (62%)	16 (89%)
**Age**	60.81 ± 9.35	49.94 ± 11.87
**Weight**	73.87 ± 12.16	71.13 ± 7.84
**Height**	1.65 ± 0.11	1.64 ± 2.12
**BMI**	27.12 ± 2.55	26.37 ± 2.12
**FPG**	93 (84, 98)	81.19 ± 8.68
**Hypertension**	4 (19%)	0 (0%)
**Total cholesterol**	196.6 ± 35.88	169.0 ± 24.55
**HDL**	60.05 (55, 84)	55.88 ± 11.57
**LDL**	104.5 ± 33.53	97.25 ± 17.94
**TG**	98.23 ± 37.93	79.19 ± 22.07
**AST**	20.56 ± 6.74	20 (17, 29)
**ALT**	18 (14, 36)	21 (10, 38)
**GGT**	32.3 ± 14.21	10.5 (8, 27)

Data are presented as the mean ± SD, median (95%CI) or number (%). BMI, body mass index; FPG, fasting plasma glucose; HDL, serum high-density lipoprotein cholesterol; LDL, serum low-density lipoprotein cholesterol; TG, serum triglycerides; AST, serum aspartate aminotransferase; ALT, serum alanine aminotransferase; GGT, gamma-glutamyl transferase.

Supplementary Table S3. List of primers used in the study.

GENE	SEQUENCE	(5'-3')
ABCA1	F	GGAGGCCAGAATGACATCTTAG
ABCA1	R	TTTCCAGCCCCATTAACTCC
PLIN2	F	AGTATCCCTACCTGAAGTCTGTG
PLIN2	R	CCCCTTACAGGCATAGGTATTG
ADIPOQ	F	ACAATGACTCCACCTTCACAG
ADIPOQ	R	TTCCTAACCGTACTGAAAGCC
ADIPOR1	F	TCATCTACCTCTCCATCGTCTG
ADIPOR1	R	CACTCAAGCCAAGTCCCAG
ADIPOR2	F	CAGCCATTATAGTCTCCCAGTG
ADIPOR2	R	CCGAGATGACATAGTGCAAGG
ANGPT1	F	TTAAAGGACTTACAGGGACAGC
ANGPT1	R	GACCACATGCATCAAACCAC
ANGPT2	F	CCACGAGACTTGAACTTCAGC
ANGPT2	R	TGTGCTTGTCTTCCATAGCTAG
ATG12	F	AATCAGTCCTTTGCTCCTTCC
ATG12	R	GCAAGTTGATTTTCTTTGTGGTTC
ATG5	F	AGCAACTCTGGATGGGATTG
ATG5	R	AGGTCTTTCAGTCGTTGTCTG
ATG7	F	TTTTGCTATCCTGCCCTCTG
ATG7	R	GCTGTGACTCCTTCTGTTTGAC
ATGL	F	CACTTCAACTCCAAGGACGAG
ATGL	R	CTCATAGAGTGGCAGGTTGTC
BGN	F	TGGAGAACAGTGGCTTTGAAC
BGN	R	GTTGTGGTCTAGGTGGAGTTC
CD14	F	CAGAGGTTCGGAAGACTTATCG
CD14	R	TTCGGAGAAGTTGCAGACG
CD206 / MRC1	F	GCAAAGTGGATTACGTGTCTTG
CD206 / MRC1	R	CTGTTATGTCGCTGGCAAATG
CD68	F	ATGGCGGTGGAGTACAATG
CD68	R	TGGACAGCTGGTGAAAGAATG
COL1A1	F	CCCCTGGAAAGAATGGAGATG
COL1A1	R	CATCCAAACCACTGAAACCTC
COL3A1	F	ATGGTGCTCCTGGTAAGAATG
COL3A1	R	GGTCCTGTGTCTCCTTTGTC
COL4A1	F	TGTGGATCGGCTACTCTTTTG
COL4A1	R	TAGTAATTGCAGGTCCCACG
COL5A1	F	TCGCTTACAGAGTCACCAAAG
COL5A1	R	GTTGTAGATGGAGACCAGGAAG
COL6A1	F	AGGAGTCAAAGGAGCAAAGG
COL6A1	R	GCATTCACAGCAAGAGCAC
COL6A3	F	AACCATTGGCTCTCACTGAAACA
COL6A3	R	GCACAGCTTTTGGTGTTTGGA
DGAT2	F	TCCGAATGCCTGTGTTGAG
DGAT2	R	CAAATAGTCTATGGTGTCCCGG
ELN	F	CCTGGCTTCGGATTGTCTC
ELN	R	CAAAGGGTTTACATTCTCCACC
F13A1	F	ATCCCATCAAAGTCAGCCG
F13A1	R	ATGTCAACGCTTCCAGTCC
FABP4	F	CATGTGCAGAAATGGGATGG
FABP4	R	AACTTCAGTCCAGGTCAACG
FASN	F	CAGAGTCGGAGAACTTGCAG
FASN	R	GGAGGCATCAAACCTAGACAG
FN1	F	GTGGCAGAAGGAATATCTCGG
FN1	R	GAGAATACTGGTTGTAGGACTGG
HAS1	F	CTTGTCAGAGCTACTTCCACTG
HAS1	R	CGGTCATCCCCAAAAGTACAG
HAS2	F	CTCAATTTTGGAAACTGCCCG
HAS2	R	CTCAGGATACATAGAAACCTCTCAC
HIF1A	F	AAGAACTTTTAGGCCGCTCA
HIF1A	R	CAACCCAGACATATCCACCTC
HSL / LIPE	F	TCATCTCCATCGACTACTCCC
HSL / LIPE	R	AGATTCGTTCCCCTGTTGAG
HYAL1	F	GATGGCTGTGGAGTTCAAATG
HYAL1	R	CCCAGAGTGCATTAGGTTCTC
HYAL2	F	TGTGAGCTTCCGTGTTCAG
HYAL2	R	CTCGCCAATGGTAGAGATGAG
IL1B	F	GGTACATCAGCACCTCTCAAG
IL1B	R	CACATTCAGCACAGGACTCTC
IL6	F	CAACCTGAACCTTCCAAAGATG
IL6	R	ACCTCAAACTCCAAAAGACCAG
IRS1	F	TCTGCTCAGCGTTGGTG
IRS1	R	GTGCATGCTCTTGGGTTTG
ITGB2	F	CCCCAAGTTTGCTGAGAGTTAG
ITGB2	R	TGAGGGCGGAAAATAACTGG
KLB	F	CATGGGTATGGGACAGGTATG
KLB	R	TCTGATGTGGGCGGAAATG
LEP	F	GCTTCAGGCTACTCCACAG
LEP	R	CCTTCCCTTAACGTAGTCCTTG
LEPR	F	TCAACCAGTACAATCCAGTCAC
LEPR	R	TTTGGGCTCAGATATGGGATG

GENE	SEQUENCE	(5'-3')
LOX	F	ACATTCGCTACACAGGACATC
LOX	R	TTCCCACTTCAGAACACCAG
LOXL2	F	GTGCAGCGACAAAAGGATTC
LOXL2	R	GCGGTAGGTTGAGAGGATG
LOXL4	F	GTGGCAGAGTCAGATTTCTCC
LOXL4	R	TTGTTCCTGAGACGCTGTTC
LPL	F	GGACTGAGAGTGAAACCCATAC
LPL	R	GGAAGGAGTAGGTCTTATTTGTGG
MCP1 / CCL2	F	CCTCCAGCATGAAAGTCTCTG
MCP1 / CCL2	R	TCTGCACTGAGATCTTCCTATTG
MMP13	F	GGAATTGGTGATAAAGTAGATGCTG
MMP13	R	ACGCGAACAATACGGTTACTC
MMP14	F	GGATGGACACGGAGAATTTTG
MMP14	R	TTTATCAGGAACAGAAGGCCG
MMP15	F	CCCAAGCCCATCAGTGTC
MMP15	R	CGCTCATTGTCGAATTTCCAG
MMP2	F	ACCCATTTACACCTACACCAAG
MMP2	R	TGTTTGCAGATCTCAGGAGTG
MMP9	F	CGAACTTTGACAGCGACAAG
MMP9	R	CACTGAGGAATGATCTAAGCCC
MOGAT1	F	GAAAGCCATCCACACTGTTG
MOGAT1	R	GCCATACTTTCCTTTGTGTTCC
MSR1	F	ATCTGTGAAATTTGATGCTCGC
MSR1	R	CCAATGAGAGGGATGAGAACTG
NPY1R	F	GATCTGAGCAGGAGAAATACCAG
NPY1R	R	CTGAATTCTTCATTCCCTTGAACTG
p16 / CDKN2A	F	GATGTCGCACGGTACCTG
p16 / CDKN2A	R	TCTCTGGTTCTTTCAATCGGG
P21 / CDKN1A	F	GAACTTCGACTTTGTCACCGAGAC
P21 / CDKN1A	R	TGGAGTGGTAGAAATCTGTCATGCT
p53 / TP53	F	CAGCACATGACGGAGGTTGT
p53 / TP53	R	TCATCCAAATACTCCACACGC
PAI-1 / SERPINE1	F	GTGGACTTTTCAGAGGTGGAG
PAI-1 / SERPINE1	R	GAAGTAGAGGGCATTCACCAG
PDGFRA	F	TTCCTCTGCCTGACATTGAC
PDGFRA	R	GTCTTCAATGGTCTCGTCCTC
PDGFRB	F	ATGTGACGGAGAGTGTGAATG
PDGFRB	R	GCAGCTCAGCAAATTGTAGTG
PLIN1	F	CATTGAGAAGGTGGTGGAGTAC
PLIN1	R	GTGTATCGAGAGAGGGTGTTG
PLIN2	F	AGTATCCCTACCTGAAGTCTGTG
PLIN2	R	CCCCTTACAGGCATAGGTATTG
PPARG	F	GTCGGTTTCAGAAATGCCTTG
PPARG	R	GCTGGTCGATATCACTGGAG
PPARGC1A / PGC1A	F	CAGGCAGTAGATCCTCTTCAAG
PPARGC1A / PGC1A	R	TCCTCGTAGCTGTCATACCTG
RPL6 F	F	CCTTAATTCTCTTTCCCATCTTGC
RPL6 R	R	TTCTTGGCTTCGGGTTTCTT
SLC2A1 / GLUT1	F	TCATCGTGGCTGAACTCTTC
SLC2A1 / GLUT1	R	GATGAAGACGTAGGGACCAC
SLC2A4 / GLUT4	F	ACTGGACGAGCAACTTCATC
SLC2A4 / GLUT4	R	GAGGACCGCAAATAGAAGGAA
SREBF1	F	TTCTGACAGCCATGAAGACAG
SREBF1	R	CCGCATCTACGACCAGTG
TCF21	F	ATACGAGAACGGGTACATTCAC
TCF21	R	CTCGCTCCAGGTACCAAAC
TGFB1	F	TTGATGTCACCGGAGTTGTG
TGFB1	R	GTAGTGAACCCGTTGATGTCC
TIMP1	F	CCCCTAGCGTGGACATTTATC
TIMP1	R	AACAGGATGCCAGAAGCC
TIMP2	F	CCCTCTGTGACTTCATCGTG
TIMP2	R	GAGATGTAGCACGGGATCATG
TIMP3	F	CCTTCTGCAACTCCGACATC
TIMP3	R	GCCTCGGTACATCTTCATCTG
TNFA	F	AGGTCTACTTTGGGATCATTGC
TNFA	R	GAAGAGGTTGAGGGTGTCTG
UCP1	F	GGACTACTCCCAATCTGATGAG
UCP1	R	AAATCCAGCGATAAGAGCCG
UCP2	F	TCCTGAAAGCCAACCTCATG
UCP2	R	GGCAGAGTTCATGTATCTCGTC
UCP3	F	AGAAAATACAGCGGGACTATGG
UCP3	R	CTTGAGGATGTCGTAGGTCAC
VEGFA	F	AGTCCAACATCACCATGCAG
VEGFA	R	TTCCCTTTCCTCGAACTGATTT
VEGFB	F	CTTAGAGCTCAACCCAGACAC
VEGFB	R	ACCCTGCTGAGTCTGAAAAG
VEGFR1	F	TCCCTCAACCTACAATCAAGTG
VEGFR1	R	GCTCTCAATTCTGTTTCCCATG
VEGFR2	F	CATTTCAAAGGAGAAGCAGAGC
VEGFR2	R	GAGGAATGGCATAGACCGTAC

F, forward; R, reverse.

Supplementary Table S4. Summary of multivariate regression models predicting TBWL.

Model	Predictor	β	SE	95% CI	p	Adjusted R^2	p
	Constant	29.394	1.539	(26.35, 32.44)	**<0.001**		
	Type of BS	4.387	1.365	(1.69, 7.09)	**0.002**		
	Age	-0.128	0.061	(-0.25, -0.01)	**0.038**		
1	Sex	-0.045	1.369	(-2.75, 2.66)	0.974	0.134	**<0.001**
	BMI	0.079	0.125	(-0.17, 0.33)	0.529		
	T2D	-2.543	1.347	(-5.21, 0.12)	0.061		
	Constant	25.888	2.085	(21.75, 30.03)	**<0.001**		
	Type of BS	6.689	1.749	(3.21, 10.17)	**<0.001**		
	Age	-0.192	0.075	(-0.34, -0.04)	**0.012**		
2	Sex	1.595	1.619	(-1.62, 4.81)	0.327	0.228	**<0.001**
	BMI	0.06	0.153	(-0.25, 0.37)	0.697		
	T2D	-1.614	1.567	(-4.73, 1.5)	0.306		
	COL1A1	-5.501	1.909	(-9.3, -1.71)	**0.005**		
	Constant	26.41	2.048	(22.33, 30.49)	**<0.001**		
	Type of BS	5.424	1.827	(1.79, 9.06)	**0.004**		
	Age	-0.146	0.076	(-0.3, 0.01)	0.059		
3	Sex	2.293	1.593	(-0.88, 5.47)	0.154	0.306	**<0.001**
	BMI	0.145	0.155	(-0.16, 0.45)	0.353		
	T2D	-1.659	1.542	(-4.73, 1.41)	0.286		
	COL5A1	-24.732	5.58	(-35.84, -13.62)	**<0.001**		
	Constant	27.128	1.791	(23.57, 30.69)	**<0.001**		
	Type of BS	6.015	1.603	(2.83, 9.2)	**<0.001**		
	Age	-0.167	0.069	(-0.3, -0.03)	**0.018**		
4	Sex	1.434	1.48	(-1.51, 4.38)	0.335	0.301	**<0.001**
	BMI	0.095	0.142	(-0.19, 0.38)	0.503		
	T2D	-2.301	1.437	(-5.16, 0.56)	0.113		
	COL6A3	-12.712	2.741	(-18.16, -7.26)	**<0.001**		

Type of BS: 0-SG, 1-GBP; sex: 0-male: 1-female; T2D: 0-no; 1-yes. β: Beta coefficient; SE: standard error; CI: confidence interval.

Supplementary Figure S1. Adipocyte size distribution across weight loss tertiles.

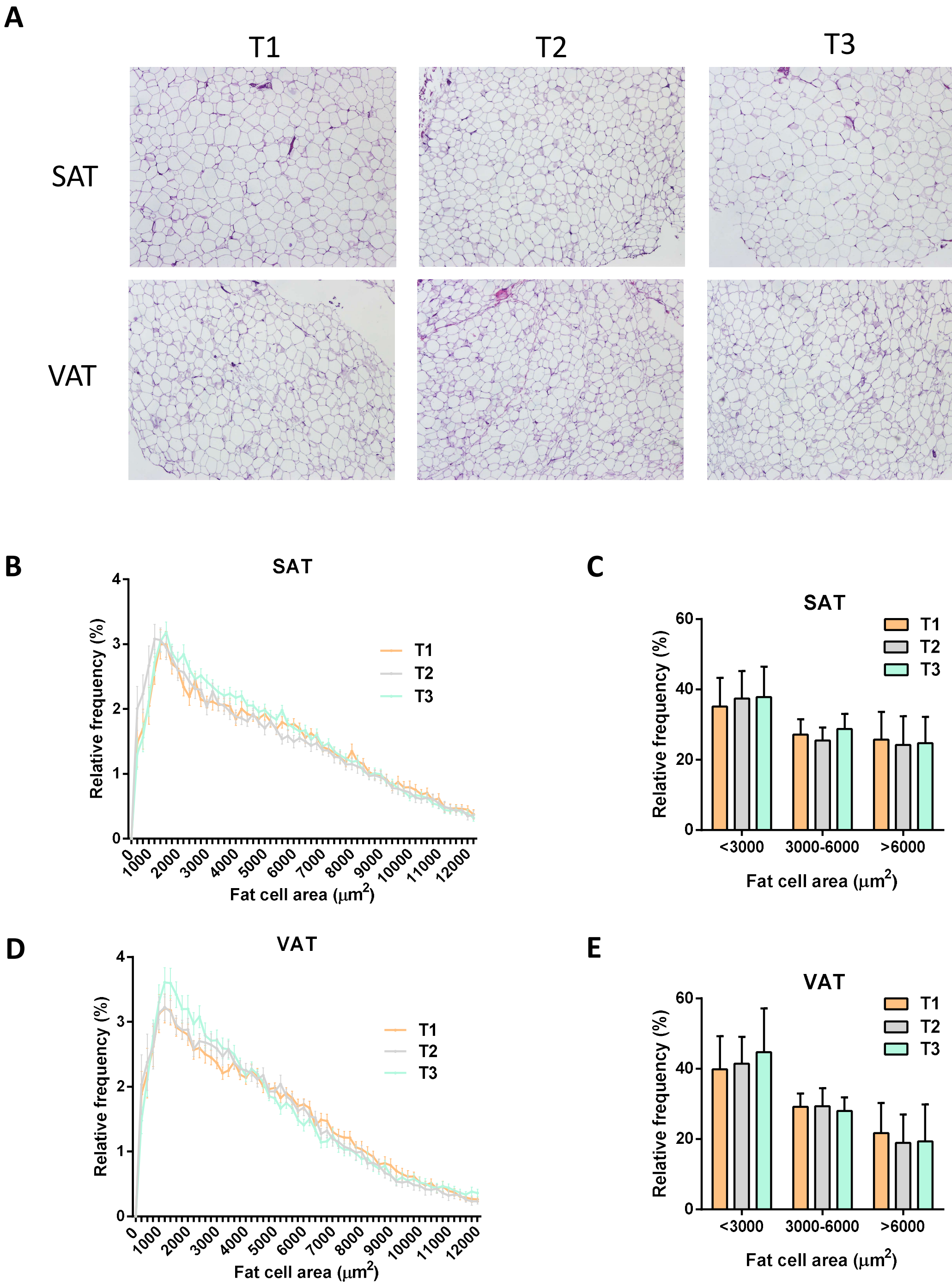

A: Representative images of hematoxylin and eosin staining of SAT and VAT samples among TBWL tertile groups. B, C: Frequency distribution analysis of fat cell areas divided by size into bin intervals of 200 μm^2 (B) and three representative sizes in SAT (C). D, E: Frequency distribution analysis of fat cell areas divided by size into bin intervals of 200 μm^2 (D) and three representative sizes in VAT (E). Data are presented as the mean ± SD.

Supplementary Figure S2. Adipose tissue fibrosis across weight loss tertiles.

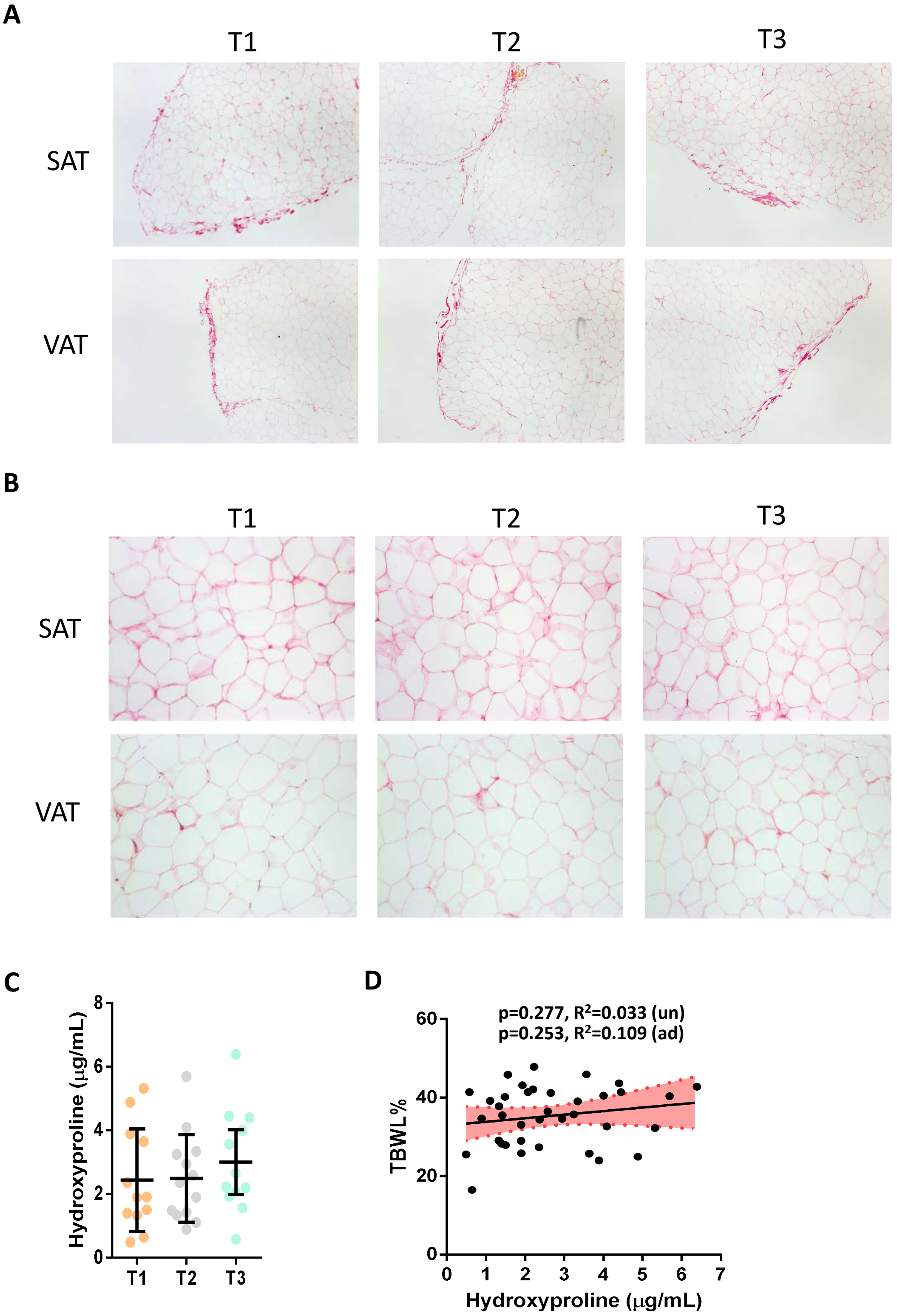

A, B: Representative images of Sirius red staining showing total (A) and pericellular fibrosis (B) in SAT and VAT samples among TBWL tertile groups. C: Determination of SAT hydroxyproline residues in TBWL tertiles. Data are presented as the mean ± SD. D: Regression analysis between hydroxyproline concentration in SAT and TBWL%. Simple linear regression (solid line) and 95% confidence interval (dashed lines) are shown. P-values are unadjusted (un) or adjusted for sex, age, baseline BMI, T2D and type of surgery (ad).
